# Cardiovascular and Ocular Parameter Alterations in Response to Cold Pressor Test in Young Adults

**DOI:** 10.3390/diagnostics14182010

**Published:** 2024-09-11

**Authors:** Adithep Daradas, Supaporn Kulthinee, Tichanon Promsrisuk, Pemika Kesornwanichwattana, Phimchanok Thaingkrathok, Sureeporn Pongampai, Pongnugoon Kongjaidee, Nutthakan Seeja, Montatip Poomvanicha, Phatiwat Chotimol

**Affiliations:** 1 Department of Optometry, Faculty of Allied Health Sciences, Naresuan University, Phitsanulok 65000, Thailand; adithepd@nu.ac.th (A.D.); pemikak58@gmail.com (P.K.); phimchanokt58@gmail.com (P.T.); sureeporn.p13@gmail.com (S.P.); pongnugoonk@nu.ac.th (P.K.); natthakans@nu.ac.th (N.S.); 2 Interdisciplinary Health and Data Sciences Research Unit (IHaDS), Faculty of Allied Health Sciences, Naresuan University, Phitsanulok 65000, Thailand; montatippo@nu.ac.th; 3 Division of Nephrology and Hypertension, Vanderbilt University Medical Center, Nashville, TN 37232, USA; skultini@gmail.com; 4 Division of Physiology, School of Medical Science, University of Phayao, Phayao 56000, Thailand; tichanon.pr@up.ac.th; 5 Department of Cardio-Thoracic Technology, Faculty of Allied Health Sciences, Naresuan University, Phitsanulok 65000, Thailand

**Keywords:** cold pressor test, cardiovascular parameters, ocular parameters

## Abstract

The sympathetic nervous responses to cold stress are known; however, concurrent cardiovascular and ocular parameter alterations in the responses are poorly characterized. The aim of this study was to examine the influence of the cold pressor test (CPT) on cardiovascular and ocular parameters in young adult subjects. There was a total of 86 participants. The CPT was conducted by submerging each participant’s left hand in cold water (3–5 °C) for 3 min. During the CPT, systolic blood pressure, diastolic blood pressure, mean arterial pressure (MAP), and heart rate were found to be significantly increased compared to the baseline and significantly decreased compared to recovery, including the mean of the standard deviations of all normal-to-normal intervals (SDNN). In the recovery phase, the SDNN continued to decrease statistically significantly compared to the baseline and the CPT. Furthermore, the findings of this study show that the CPT impacted intra-ocular pressure (IOP), ACD, and pupil size parameters. There was a positive correlation between the MAP and IOP in both eyes during the CPT. The cold stress stimulates a sympathetic response, leading to an increase in the MAP. The pupil size increased in response to the CPT in both eyes, indicating that ocular function was increased in response to the CPT in young adults compared to baseline. In conclusion, our results suggest that in young adults, cardiovascular and ocular parameters respond to the sympathetic nervous system during the CPT.

## 1. Introduction

The autonomic nervous system (ANS) consists of two antagonistic sets of nerves, the parasympathetic nervous system (PNS) and the sympathetic nervous system (SNS), which regulate involuntary body functions [1]. The ANS plays an important part in maintaining the overall physiological state of an organism when it experiences changes in its environment [2]. Despite the apparent dissimilarity between the heart and eyes, the ANS regulates both of them [3,4]. The stimulation of the SNS elicits an increase in heart rate and myocardial contractility, as well as blood vessel constriction and pupil dilation [5,6]. Because the diameter of blood vessels is regulated by SNS input, the SNS plays an important role in regulating the balance between intraocular pressure and ocular blood flow [7].

The cold pressor test (CPT) is a procedure that employs a potent and standardized stressor to elicit a cardiovascular reaction [8]. The CPT involves submerging a subject’s hand in ice water. The cold stimulates a sympathetic response, leading to an elevation in blood pressure [9,10]. Stress triggers the activation of the sympathetic function of the ANS, leading to a rise in heart rate and cardiac output [11,12,13]. Reports indicate that ocular circulation can respond autonomously to variations in blood pressure [14].

Our previous study showed that the arteriosclerosis index increased during CPT in normal-weight and overweight participants [15]. Previous studies [16,17] have demonstrated the application of the CPT to study ocular conditions. During cold stimulation, the pupil diameter increased significantly, and the IOP decreased [7]. However, previous studies have reported that the IOP increased during the CPT [18,19]. Reports indicate that autoregulation in the ocular system did not effectively prevent an increase in the choroidal vessel blood flow velocity during the CPT [20]. This indicates that a sympathoexcitatory stimulus in response to the CPT affects the ocular condition. However, the potential effects of CPT on the cardiovascular and concurrent ocular parameters during the cold pressor response remain unclear. We hypothesize that both cardiovascular and ocular parameters will change as a result of the sympathetic response to the CPT in young adults.

## 2. Materials and Methods

### 2.1. Research Participants

Eighty-six young adult volunteers between the ages of 20 and 40, both male and female, were included in this study, which was conducted in January and February 2020. The research methodology received approval from the Naresuan University Institutional Review Board (approval certificate number 0675/62) after all participants provided informed written consent. All volunteers completed a screening medical questionnaire prior to their participation. This questionnaire was designed to identify factors that could potentially confound the measurement of the study parameters. Individuals reporting a history of smoking, alcohol consumption, ocular infections, glaucoma, diabetes, cardiovascular disease, or vascular disorders were excluded from the study.

### 2.2. Experimental Methodology

#### 2.2.1. Cold Pressor Test

The cold pressor test was performed as described in a previous study [21]. Briefly, the cold stress test was conducted by immersing the left hand up to the wrist, palm facing down, into a container filled with ice-cold water while the participant remained seated. The water temperature was carefully controlled using a thermometer, ensuring it remained within 3 to 5 degrees Celsius. Additionally, the water temperature was continuously monitored throughout the experiment. If the volunteer experienced unbearable cold or pain, they could withdraw from the experiment at any time, and their participation was discontinued. As a baseline, the cardiovascular parameters, including blood pressure, heart rate, heart rate variability indices, and ocular parameters of the participants were monitored prior to the CPT. These measurements were carried out twice: once for the duration of the CPT and once for four minutes during recovery.

#### 2.2.2. Assessment of Blood Pressure

The blood pressure was measured after five to ten minutes of rest using calibrated oscillometric equipment (Omron M7^®^, Omron Corporation, Kyoto, Japan). The blood pressure readings were measured three times, and the average values for systolic blood pressure (SBP), diastolic blood pressure (DBP), mean arterial pressure (MAP), and heart rate (HR) were computed from these measurements. The pulse pressure (PP) is the numerical value obtained by subtracting the DBP from the SBP. The MAP was determined using the formula [(2 × DBP) + SBP]/3 [22].

#### 2.2.3. Heart Rate Variability

The participants were instructed to lie in a supine position in a quiet room (room temperature: 25 °C), relax, and breathe spontaneously at their own rate. Each participant was asked to keep as still as possible throughout the recording period. Then, baseline HRV measurements were obtained after 5 min of resting at the start of the study. Subsequently, the participants were asked to immerse their left hand in cold water for a period of 3 min of cold stimulation, followed by removing the hand from the water and the continuation of HRV recording for another 4 min of recovery.

The HRV was measured through an autoregressive power spectral analysis of the R-R interval in lead 2 of the electrocardiogram (EDAN SE-1515, Edan Instruments, Shenzhen, China). Two methods were used for the assessment: statistical operations on R-R intervals for the time domain analysis, and spectral analysis on a series of R-R intervals for the frequency domain analysis. The time domain parameters used to assess the HRV included the standard deviation of all NN intervals (SDNN), indicating the combined influence of the sympathetic and parasympathetic branches of the autonomic nervous system on the heart rate, and the square root of the mean of the sum of the squares of differences between adjacent NN intervals (RMSSD), which serves as an indicator of parasympathetic activity. The frequency domain parameters included the low-frequency spectral power (LF) (0.04–0.15 Hz), reflecting the combined effects of the vagal and sympathetic components on the heart, with a predominant sympathetic influence, and the high-frequency spectral power (HF) (0.15–0.4 Hz), indicating the influence of the vagus nerve on heart activity. The LF/HF ratio reflects the balance between sympathetic and parasympathetic nervous system activity in the heart [23].

#### 2.2.4. Ocular Parameter Measurements

Ocular parameters were measured noninvasively. The IOP measurement was conducted using a non-invasive tonometer (Canon TX-20P, Canon Inc., Tokyo, Japan). The assessment of the anterior segmental parameters of the eye, such as the anterior chamber depth (ACD), anterior chamber angle (ACA), pupil size, and central corneal thickness (CCT), was conducted using optical coherence tomography (OCT) (CIRRUS HD-OCT 5000, Carl Zeiss Meditec AG, Jena, Germany).

### 2.3. Analytical Statistics

The statistical analysis was conducted using SPSS for Windows, version 17.0 (SPSS, Inc., Chicago, IL, USA). A *p*-value of less than 0.05 was considered statistically significant. The Kolmogorov–Smirnov test was employed to confirm the normality of the data distribution. The variables compared to the baseline, CPT, and recovery were analyzed using repeated-measures analysis of variance (ANOVA) and pairwise comparisons with Bonferroni correction. The correlation between the two variables was determined using linear regression analysis.

## 3. Results

### 3.1. Characteristics of Participants

Eighty-six participants were included in this study. The participants had a mean age of 22.84 ± 2.50, and 61.63% were women. According to the results of the questionnaire, all subjects were free of heart disease and dyslipidemia. Among the participants, 12.79% had allergies, and 1.16% had hypertension and asthma. Most of the subjects (95.35%) did not have ocular diseases; of the remaining participants, 2.32% had eye allergies, 1.16% had dry eyes, and 1.16% had color blindness. Descriptive characteristics of the study sample can be found in Table 1.

### 3.2. Cardiovascular Responses to CPT

**Figure 1:**
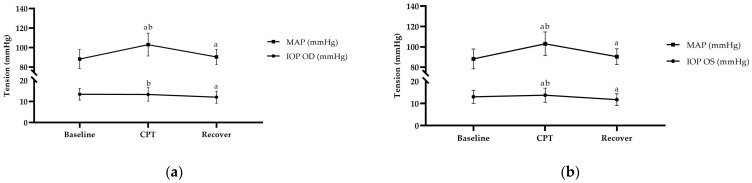
The repeated-measures ANOVA; Bonferroni post hoc test; ^a^ *p* < 0.05 versus baseline; ^b^ *p* < 0.05 versus recovery: (**a**) MAP and IOP (OD) response to CPT; (**b**) MAP and IOP (OS) response to CPT.

During the CPT, the SBP, DBP, and MAP (Figure 1a,b) were significantly increased (*p* < 0.001) after the hand’s immersion in ice water. The HR also showed significant enhancement (*p* < 0.001) in response to the CPT. Within 2 min after the CPT, the blood pressure (BP) and HR gradually decreased but did not return to baseline values (Table 2).

### 3.3. HRV Responses to CPT

HRV measurements obtained before, during, and after the cold pressor test are presented in Table 2. The SDNN decreased from 50.88 ± 21.26 ms at the baseline to 48.17 ± 21.23 during the CPT, but the difference was not statistically significant. During the recovery phase, the SDNN continued to decrease to 43.65 ± 19.98, which is statistically significant compared to the baseline. However, there were no statistical significances in the RMSSD, LF, HF, or LF/HF ratio.

### 3.4. Ocular Responses to CPT

The baseline ocular parameters are shown in Table 3, with the IOP, ACD, ACA, pupil size, and CCT showing right eye (OD) and left eye (OS) averages of 13.48 ± 2.77 and 13.01 ± 2.97 mmHg, 3.04 ± 0.28 and 3.02 ± 0.29 mm, 36.64 ± 5.80 and 36.69 ± 5.23 degrees, 3.02 ± 0.84 and 3.11 ± 0.89 mm, and 534.01 ± 29.38 and 536.27 ± 30.09 µm, respectively. The IOP in the OD was significantly decreased during recovery and was not significant during the CPT, but in the OS, it was significantly increased during the CPT and decreased afterward. The ACD was increased in the OD significantly during the CPT and recovery and in the OS significantly during recovery. Additionally, the pupil size in both the OD and OS was significantly increased during the CPT and returned to baseline during recovery. However, no significant changes in the ACA or CCT occurred during the CPT when compared to the baseline, while all ocular data recovered to their initial levels after the CPT (Table 3).

### 3.5. Correlation between MAP and IOP

**Figure 2:**
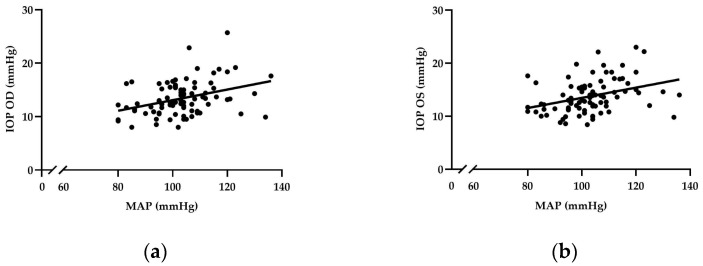
Linear regression analyses of the MAP and IOP. There was a significant correlation during the CPT: (**a**) correlation between the MAP and IOP (OD); (**b**) correlation between the MAP and IOP (OS).

According to a univariate analysis, there was a positive relationship between the MAP and IOP in the OD and OS at the baseline (b = 0.083, *p* = 0.006 and b = 0.082, *p* = 0.012, respectively). In addition, the results show a positive correlation between the MAP and IOP in the OD and OS during the CPT (b = 0.099, *p* = 0.001 and b = 0.096, *p* = 0.001, respectively) (Figure 2a,b). Moreover, a positive correlation was found between the MAP and IOP in the OD during recovery (b = 0.108, *p* = 0.006). Furthermore, after adjusting for age and BMI, there was a positive correlation between the MAP and IOP in both eyes at the baseline and during the CPT. However, no significant correlation was found during the recovery phase, as shown in Table 4.

## 4. Discussion

The CPT is a common method for activating the sympathetic nervous system and is performed by placing a subject’s hand in a container of cold water, inducing slow pain that is terminated by immediately removing the hand from the cold water. A cardiac autonomic function can be evaluated by monitoring the cardiovascular parameters, such as the BP, HR, HRV, and other hemodynamic parameters. Our results show that cutaneous cold led to a significant increase in the HR and BP compared to the resting conditions. Cold stimuli activate the afferent sensory pathways (via the periphery stimulation), which triggers a vascular sympathetic response and increases peripheral resistance, resulting in an increase in the BP; this is typically accompanied by an increase in the HR [9,24].

From our data, the CPT induced an average increment in the SBP of 13.87 mmHg and an increase of 14.08 mmHg in the DBP. In 2021, Lamotte et al. defined a normal response to the CPT as an increment in the SBP of less than 20 mmHg, whereas subjects whose SBP increased by at least 25 mmHg or whose DBP increased by at least 20 mmHg were considered ‘hyperreactive’ [25]. Cold stress activates cutaneous cold receptors, which then transmit signals to afferent sensory pathways, triggering a sympathetic response [25]. Local cold stress causes vasoconstriction mainly through noradrenaline released from the sympathetic nerves and subsequently bound to *α*-adrenergic receptors, resulting in an increase in the systolic and diastolic blood pressure [26].

Measuring the HRV has been considered a useful noninvasive technique to detect the function of the autonomic nervous system. It is an indirect indicator of cardiac autonomic modulation since the HR is under the dual control of direct sympathetic and parasympathetic innervation at the SA node, but it is influenced by sympathoadrenal activation and also influenced by local reflexes [27].

Our present study investigated the effect of the CPT on HRV indices. After the CPT, the sympathetic nervous system indicator SDNN decreased and continued to significantly decrease during the recovery phase, which reflects sympathetic overactivity. Meanwhile, no changes in the frequency domains of the LF, HF, or LF/HF ratio were observed. Interpretation of the HRV is complicated because the modulation mechanism is complex and depends on many factors. These include not only autonomic nerve activity but also mechanical and hemodynamic influences, whereby the activity of one branch can, in some instances, augment, and in other instances, attenuate the activity of the other branch. Conflicting results in the literature can occur because of variations in terms of the participants and CPT protocol, including various HRV procedures and analysis methodologies.

This study revealed a positive correlation between the MAP and IOP in both eyes during the CPT. Furthermore, the multiple regression analysis was used to adjust the confounding factors of age and BMI. The MAP remained associated with an elevated IOP [28,29]. Similarly, there was a report of a positive correlation between the MAP and IOP in healthy Korean men [30]. In contrast, there was a strong negative association between the MAP and IOP in primary open-angle glaucoma patients [31]. However, the mechanism of the relationship between the IOP and MAP remains unclear. In a previous study, it was reported that fluctuations in BP can lead to fluctuations in ciliary blood pressure, affecting the ability of the aqueous humor to filter particles and causing changes in the IOP [32]. A positive correlation between elevated SBP and DBP is associated with a higher incidence of ocular hypertension [33]. This relationship is further affirmed by our study, which indicates that elevated SBP, DBP, and MAP are indicative of sympathetic hyperactivity. These elevated pressure parameters contribute to an increase in the IOP, suggesting that a heightened cardiovascular response is a contributing factor to ocular disorders. Moreover, sympathetic reactivity is shown to influence the IOP in both groups. In contrast, the OD results of the normal-weight group align with the findings of Chen et al. 2019, who discovered that the IOP decreased under the CPT and reported that parasympathetic and sympathetic innervation influenced the generation and outflow of aqueous humor, although it remains unclear exactly what mechanism underlies any observed alterations in the IOP [7].

Accordingly, the IOP causes mechanical stretching of the lamina cribrosa and damage to the retinal ganglion cells. Therefore, elevated IOP is crucial for the development of glaucoma. Glaucoma is a progressive optic neuropathy characterized by a loss of optic nerve fibers and retinal ganglion cells, and it is a major cause of severe eye disease and blindness [34,35]. Generally, pupil size and changes in pupil size reflect the balance between the sympathetic (pupil dilation) and parasympathetic (pupil constriction) nervous systems [36]. In addition, pupil size and its changes are influenced by certain variables, including time, light, environment, sleepiness, and emotional state [18]. In our study, an increased pupil size during the CPT was found in both eyes, and an increased ACD was found in the OD. Kiel et al., 2022, suggested an association between increased pupil size and a deeper anterior chamber [37]. Furthermore, previous studies have reported pupil dilation and changes during the CPT in both healthy German subjects and healthy Chinese participants [7,18]. Bullock et al., 2022, also observed that CPT caused rapid pupil dilation within 10 s of exposure [38]. Nonetheless, eyes with larger pupils often had lower visual acuity (VA), while those with smaller pupils exhibited higher VA [39,40]. This indicates that a person’s vision may be affected by pupil dilation. Therefore, sympathetic hyper-reactivity in response to the CPT should be considered in a concomitant ocular examination.

This study has various limitations. The health of the volunteers was assessed solely through questionnaires without medical examinations or biological laboratory results. Furthermore, this study was constrained by a small sample size. Also, this study focused on the age range of young adults, which limits the generalizability of the findings to other age groups. Future studies should seek to include more diverse participants, large populations, and diseases to strengthen the external validity of the findings.

## 5. Conclusions

In conclusion, our study revealed a significant increase in cardiovascular parameters at the baseline condition, which could be due to sympathetic overactivity in the young adult participants, as evidenced by a reduced SDNN that continued to significantly decrease during the recovery phase. There was a positive correlation between the MAP and IOP in both eyes during the CPT. Cold stress stimulates a sympathetic response, leading to an increase in the MAP. It is possible that high MAP could result in elevated IOP during the CPT. The pupil size increased in response to the CPT in both eyes. Cardiovascular and ocular parameters were more hyper in response to the CPT in young adults compared to baseline.

## Figures and Tables

**Table 1:** Characteristics of the study participants.

Characteristics	Total (*n* = 86)
Age, (year)	22.84 ± 2.50
BMI, (kg/m^2^)	22.93 ± 3.69
Sex	
Male	33 (38.37%)
Female	53 (61.63%)
History of illness	
No	73 (84.88%)
Yes	13 (15.12%)
Cardiovascular disease	0
Dyslipidemia	0
Allergy	11 (12.79%)
Hypertension	1 (1.16%)
Asthma	1 (1.16%)
Ocular diseases	
No	82 (95.35%)
Yes	4 (4.65%)
Glaucoma	0
Eye infection	0
Allergy to eyes	2 (2.32%)
Dry eye syndrome	1 (1.16%)
Color blindness	1 (1.16%)

BMI: body mass index. Values are expressed as mean ± SD, number of participants, and percentage of participants.

**Table 2:** Cardiovascular parameter changes during and after CPT.

Parameters	Baseline	CPT	Recovery	*p*-Value
SBP (mmHg)	119.27 ± 12.66	133.14 ± 16.37 ^ab^	121.29 ± 11.55 ^a^	<0.001 *
DBP (mmHg)	71.05 ± 8.42	85.13 ± 10.00 ^ab^	73.42 ± 6.74 ^a^	<0.001 *
MAP (mmHg)	88.19 ± 9.73	102.98 ± 11.62 ^ab^	90.33 ± 7.81 ^a^	<0.001 *
PP (mmHg)	48.22 ± 9.83	48.01 ± 10.60	47.87 ± 8.33	0.932
HR (bpm)	76.45 ± 10.75	84.12 ± 12.35 ^ab^	76.36 ± 10.87	<0.001 *
SDNN (ms)	50.88 ± 21.26	48.17 ± 21.23 ^b^	43.65 ± 19.98 ^a^	<0.001 *
RMSSD (ms)	45.50 ± 24.89	43.94 ± 25.10	44.15 ± 23.03	0.700
LF (norm)	51.64 ± 30.98	46.51 ± 20.21	46.59 ± 19.75	0.236
HF (norm)	48.65 ± 31.93	53.49 ± 20.21	53.41 ± 19.75	0.272
LF/HF	1.65 ± 1.80	1.44 ± 2.29	1.22 ± 1.06	0.242

* Statistically significant according to repeated-measures ANOVA; Bonferroni post hoc test; ^a^ *p* < 0.05 versus baseline; ^b^ *p* < 0.05 versus recovery. The values are expressed as the mean ± SD. CPT: cold pressure test; SBP: systolic blood pressure; DBP: diastolic blood pressure; MAP: mean arterial pressure; PP: pulse pressure; HR: heart rate; SDNN: mean of the standard deviations of all normal-to-normal (NN) intervals; RMSSD: root mean square of the sum of the differences between adjacent NN intervals; LF: low-frequency spectral power; HF: high-frequency spectral power; LF/HF: ratio of LF to HF power.

**Table 3:** Ocular parameter changes during and after CPT.

Parameters		Baseline	CPT	Recovery	*p*-Value
IOP (mmHg)	OD	13.48 ± 2.77	13.39 ± 3.18 ^b^	12.08 ± 2.86 ^a^	<0.001 *
	OS	13.01 ± 2.97	13.76 ± 3.20 ^ab^	11.74 ± 2.66 ^a^	<0.001 *
ACD (mm)	OD	3.04 ± 0.28	3.08 ± 0.26 ^a^	3.08 ± 0.26 ^a^	<0.001 *
	OS	3.02 ± 0.29	3.04 ± 0.29	3.08 ± 0.28 ^a^	0.006 *
ACA (degree)	OD	36.64 ± 5.80	35.61 ± 5.25	36.01 ± 4.29	0.097
	OS	36.69 ± 5.23	35.94 ± 5.09	35.71 ± 5.03	0.161
Pupil size (mm)	OD	3.02 ± 0.84	3.21 ± 0.77 ^ab^	3.00 ± 0.79	0.008 *
	OS	3.11 ± 0.89	3.27 ± 0.76 ^ab^	3.13 ± 0.71	0.038 *
CCT (µm)	OD	534.01 ± 29.38	535.94 ± 32.69	530.40 ± 35.31	0.078
	OS	536.27 ± 30.09	536.34 ± 31.79	535.67 ± 29.57	0.800

* Statistically significant according to repeated-measures ANOVA; Bonferroni post hoc test; ^a^ *p* < 0.05 versus baseline; ^b^ *p* < 0.05 versus recovery. The values are expressed as the mean ± SD. CPT: cold pressor test; IOP: intraocular pressure; ACD: anterior chamber depth; ACA: anterior chamber angle; CCT: central corneal thickness; OD: oculus dexter (right eye); OS: oculus sinister (left eye).

**Table 4:** Linear regression analysis enter method evaluating factors influencing the IOP, with adjusted age and BMI.

Parameters	Univariate			Multivariate		
**IOP (OD)**	**b**	**95% CI**	***p*-Value**	**b**	**95% CI**	***p*-Value**
MAP at Baseline	0.083	0.024 to 0.143	0.006 *	0.083	0.015 to 0.150	0.017 *
MAP during CPT	0.099	0.044 to 0.155	0.001 *	0.078	0.002 to 0.153	0.044 *
MAP during Recovery	0.108	0.032 to 0.184	0.006 *	0.078	−0.004 to 0.160	0.063
**IOP (OS)**	**b**	**95% CI**	***p*-Value**	**b**	**95% CI**	***p*-Value**
MAP at Baseline	0.082	0.018 to 0.146	0.012 *	0.074	0.001 to 0.147	0.048 *
MAP during CPT	0.096	0.040 to 0.152	0.001 *	0.115	0.040 to 0.190	0.003 *
MAP during Recovery	0.065	−0.007 to 0.137	0.078	0.041	−0.037 to 0.120	0.298

* Statistically significant according to linear regression analysis enter method. CPT: cold pressor test; MAP: mean arterial pressure; IOP: intraocular pressure; OD: oculus dexter (right eye); OS: oculus sinister (left eye).

## Data Availability

The datasets generated in this study are available from the corresponding author upon request.
